# “Poverty is the big thing”: exploring financial, transportation, and opportunity costs associated with fistula management and repair in Nigeria and Uganda

**DOI:** 10.1186/s12939-018-0777-1

**Published:** 2018-06-01

**Authors:** Kaji Tamanna Keya, Pooja Sripad, Emmanuel Nwala, Charlotte E. Warren

**Affiliations:** 1 Population Council, Maternal and Newborn Health, 4301 Connecticut Avenue NW Suite 280, Washington, DC 20008 USA; 2 Population Council, No. 16 Mafemi Crescent, Off Solomon Lar Way, Utako District, Abuja, Nigeria

**Keywords:** Transportation cost, Financial cost, Opportunity cost, Employment, Fistula, Barriers, Enablers

## Abstract

**Background:**

Women living with obstetric fistula often live in poverty and in remote areas far from hospitals offering surgical repair. These women and their families face a range of costs while accessing fistula repair, some of which include: management of their condition, lost productivity and time, and transport to facilities. This study explores, through women’s, communities’, and providers’ perspectives, the financial, transport, and opportunity cost barriers and enabling factors for seeking repair services.

**Methods:**

A qualitative approach was applied in Kano and Ebonyi in Nigeria and Hoima and Masaka in Uganda. Between June and December 2015, the study team conducted in-depth interviews (IDIs) with women affected by fistula (*n* = 52) – including those awaiting repair, living with fistula, and after repair, and their spouses and other family members (*n* = 17), along with health service providers involved in fistula repair and counseling (*n* = 38). Focus group discussions (FGDs) with male and female community stakeholders (*n* = 8) and post-repair clients (*n* = 6) were also conducted.

**Results:**

Women’s experiences indicate the obstetric fistula results in a combined set of costs associated with delivery, repair, transportation, lost income, and companion expenses that are often limiting. Medical and non-medical ancillary costs such as food, medications, and water are not borne evenly among all fistula care centers or camps due to funding shortages. In Uganda, experienced transport costs indicate that women spend Ugandan Shilling (UGX) 10,000 to 90,000 (US$3.00-US$25.00) for two people for a single trip to a camp (client and her caregiver), while Nigerian women (Kano) spent Naira 250 to 2000 (US$0.80-US$6.41) for transportation. Factors that influence women’s and families’ ability to cover costs of fistula care access include education and vocational skills, community savings mechanisms, available resources in repair centers, client counseling, and subsidized care and transportation.

**Conclusions:**

The concentration of women in poverty and the perceived and actual out of pocket costs associated with fistula repair speak to an inability to prioritize accessing fistula treatment over household expenditures. Findings recommend innovative approaches to financial assistance, transport, information of the available repair centers, rehabilitation, and reintegration in overcoming cost barriers.

## Background

Poverty greatly hinders women’s access to obstetric fistula repair; its effect is compounded by low socio-economic status and level of education, rural residence, lack of prenatal care, and early marriage [1, 2]. A woman living with an unrepaired fistula experiences ostracism, stigma, shame, and partner rejection, is often shunned by her community, in addition to physical consequences such as fetid odor, frequent pelvic or urinary infections, painful genital ulcerations, thigh inflammation from constant wetness, infertility, leg nerve damage, and even early mortality [3]. Obstructed labor, the primary cause of obstetric fistula, often results in newborn asphyxia, which can lead to stillbirth, brain damage, or neonatal death [4–7]. Although fistula is preventable and treatable, its prevalence in sub-Saharan Africa is at 1.57 cases per 1000 women [8]. According to the World Health Organization (WHO), each year between 50,000 and 100,000 women are affected by obstetric fistula globally [9]. Nigeria’s annual obstetric fistula incidence is 2.11 per 1000 live births, and in Uganda 2 % of women ages 15 to 49 report experience of fistula at some point in their lives [10, 11].

A recent literature review describes nine direct types of barriers to accessing fistula repair: psychosocial, social, political, financial, along with awareness, transportation, facility, and care quality [1]. Of these nine barriers, financial (85 out of 137 articles) and transportation (69 out of 137 articles) are frequently cited for delayed fistula care—or lack thereof. Opportunity cost or income loss, generally understood as the cost of lost productivity and hours separated from family, is not mentioned separately in this review, but it is evident that employment cost presents an insurmountable barrier to accessing fistula repair for those in marginal economic circumstances [12].

In low- and middle-income countries, financial barriers due to poverty, along with the high medical and non-medical costs of repair delay decisions for fistula repair [13–15]. Most African countries lack mechanisms for preventing catastrophic medical expenditures for families and households [16]. Out of pocket payment costs comprise 70% of health financing in low income countries [17]. According to WHO, in-patient care in Africa is associated with greater likelihood of borrowing and selling assets [18]. Fistula treatment is more expensive than other common illness in Africa region such as pneumonia and malaria. In Tanzania, US $5 for non-complicated malaria and in Kenya US$288 for cerebral malaria with neurological sequelae [19]. In 2014, the Fistula Foundation reported that, on average, US$450 is required to treat obstetric fistula including surgery, post-operative care, and physical rehabilitation [20]. This estimation was based on cost analyses from 25 countries in sub-Saharan Africa and South Asia. Transportation barriers such as cost, lack of frequency, distance to hospitals and duration, or no transportation at all in remote areas influence women’s access to fistula repair services [1, 8].

Access to obstetric fistula repair services continues to be a challenge in Nigeria and Uganda [21], both of which are high burden countries [10, 11]. The United States Agency for International Development (USAID) funds the two countries under Fistula Care *Plus* (FC+), five-year fistula repair and prevention project. In Nigeria, FC+ currently supports 19 prevention-focused facilities and 10 hospitals in 12 states, for awareness, prevention, treatment, and repair of obstetric fistula. In Uganda the project supports 13 sites for prevention activities and five hospitals (three private, faith-based and two government) in different districts across the county.

While Nigeria conducts routine fistula repairs at designated hospitals—national obstetric fistula centers—Uganda frequently provides camp-based fistula repair two or three times each year at specified tertiary referral hospitals. Ugandan communities receive information about repair camps by radio and through village health teams (VHTs). Despite government and donor efforts to increase availability of repair services in both countries, reported repairs are less than their actual fistula cases, generally due to obstructed labor and unmet need for emergency obstetric care [22–24]. Understanding the range of cost barriers facing these countries’ communities is important for increasing repair [17]. This study explores not only financial but transportation and opportunity cost barriers, and enabling factors, to inform interventions for addressing accessibility challenges in Nigeria and Uganda.

## Methods

### Study sites

Nigeria and Uganda were selected as priority countries for formative research, and this study was conducted in catchment areas of and at two specialized national fistula centers, in northern and southern Nigeria (Katsina and Ebonyi), and at camp sites at regional referral and faith-based hospitals in eastern and western Uganda (Hoima and Masaka) supported by the FC+ project. Geographically and linguistically distinct sites were purposively selected to capture sub-national diversity. Population Council selected these sites in consultation with Nigerian and Ugandan USAID missions, ministries of Health, the FC+ project, and Ugandan FC+ partner TERREWODE. Sites were further selected by their ability to capture diverse experiences, by women and care providers, of fistula care access, quality, treatment, and re-integration.

Nigeria’s national fistula centers span the country’s six geopolitical zones, with one center expected to serve five or six states. Our study was conducted in two of these six centers. Although fistula centers operate with support from the Federal Ministry of Health, in collaboration with development partners, they differ by scope of care provision. In Katsina, repaired women are admitted to a reintegration program housing and training post-repair clients in a trade of their choice for up to 6 months, while in other hospitals repaired women return home after 2 weeks, without prolonged rehabilitation. Although fistula repair at designated centers is free, ancillary costs such as caregiver expenses and transportation costs to distant repair centers burden poor clients. Similarly, private hospitals that provide fistula repair services, at high (out of pocket) costs to patients, impede access, in addition to poor referral services to fistula centers from primary and secondary health facilities—often clients’ first health system contacts. In general, basic maternal and reproductive healthcare in Nigeria’s public sector is free of charge as policy, although this does not always translate to practice [25, 26].

In Uganda, like Nigeria, fistula repair services are free and rely heavily upon donor funding, resulting in the dominant camp-based service model, although there are increasing policy shifts towards offering routine repairs at referral hospitals. Ugandan fistula repair sites provide various re-integration services for post-repair women. Village health teams, primary health facilities (level 2, 3, 4), and district and referral hospitals constitute Uganda’s tiered health system. Private care providers are common sources of care, both nationally and locally: 78% of non-public healthcare facilities are faith-based, for example the Uganda Catholic Medical Bureau, Uganda Protestant Medical Bureau, and Uganda Muslim Medical Bureau [27]. In general, 65% of Uganda’s healthcare services are paid out-of-pocket, with as many as 20% of Ugandans incurring catastrophic health expenditures, which can contribute to delayed fistula [27].

### Study design

Given this study’s formative and exploratory nature, a qualitative approach was best suited to understanding local perspectives of fistula repair barriers and enablers in both Nigeria and Uganda. The sample size was guided on theoretical saturation, with thematic areas fully explained by respondents. The study coordinator (from Population Council) maintained daily contact with field supervisors, who monitored data collection quality and progress, including debriefing sessions where interviewers discussed themes emerging from the data. These sessions and concurrent data management allowed the authors to assess saturation of fistula care barriers and enablers. The study team conducted in-depth interviews (IDIs) with women who experienced fistula, and their family members and service providers (*n* = 107), and facilitated focus group discussions (FGDs, *n* = 14), with eight to ten male and female community stakeholders and post-repair clients (Table 1).

### Participant selection

Participants were purposively selected based on their experience (themselves or a family member) of living with fistula in Nigeria or Uganda, or from a professional role in providing fistula repair or counseling. The study team recruited women 18 years old and older who were living with fistula, at the fistula centers (Nigeria) or camps (Uganda) awaiting repair, or during the post-repair recovery period (5 to 14 days following surgery) (Table 1). Women answered open-ended questions about their experiences before, during, and after their repair. Ugandan women and their family members stay at a repair camp prior to surgery, and this model helped the team interview more people from Uganda, to better understand their perspectives. At the Ugandan sites 17 family members who accompanied women for repair were interviewed; although primarily spouses, they included aunts, parents, and siblings. These perspectives allow both corroboration of women’s narratives as well as understanding of the experience of those escorting a fistula patient to a treatment center. The study team interviewed 38 fistula care providers in Nigeria (*n* = 11) and Uganda (*n* = 27)—nurses, midwives, counselors, surgeons, matrons, facility managers, and one policymaker—to better understand the health system contexts of these surgeries as well as the barriers and enablers described by women and their families. Community stakeholders including religious leaders, village or district heads, women’s groups, and traditional birth attendants were recruited for separate male and female FGDs (8 to 10 persons per group).

**Table 1:** Description of qualitative data collection

Type of interview	Nigeria		Uganda		Total
	Kano	Ebonyi	Hoima	Masaka	
In-depth interview	16	18	40	33	107
Women living with fistula	8	9	20	15	52
Spouse/accompanying family members	4	2	6	5	17
District managers/Providers at camps/facility	4	7	14	13	38
Focus group discussions	3	3	4	4	14
Post repair clients	1	1	2	2	6
Community stakeholders-women	1	1	1	1	4
Community stake holders-men	1	1	1	1	4

Data were collected in Nigeria and Uganda from June through December 2015. Experienced research assistants were hired and trained, and practiced interviewing and note-taking prior to data collection. Interviewers conducted IDIs and FGDs in local languages—Hausa (in Kano), Igbo (in Ebonyi), Luganda (in Masaka and Hoima), and Runyoro (in Hoima)—for better understanding and open description of respondents’ experiences. Self-reported cost questions, including what women and their families paid for transportation, care at home, alternative treatments, and medical and non-medical expenses, were asked in an open-ended format. Interviewers briefed potential interviewees on the study’s purpose, its voluntary nature, and the risks and benefits of participation prior to participants’ voluntary informed consent. Respondents received no monetary incentive for participating but were reimbursed (USD$6) for transportation costs, and were offered refreshments during FGDs.

### Data analysis

Interviews and discussions were audio-recorded, transcribed, and translated into English by local translators in Nigeria and Uganda. Multiple analysts (at least two individuals) read each document for content. A codebook was inductively derived based on analysts’ discussions of their analyses, with emerging codes identified. The final code structure was applied to all data with NVivo 11 and Atlas.ti software. Subsequent analytic memos were written on emergent themes and participant perspectives developed into the barrier and enabler domains on transportation, financial, and opportunity cost factors influencing access to services. Self-reported costs presented reflect both typical responses by women and communities; in some instances, when relevant, a range was provided to describe variability within and across settings.

### Ethical approval

Ethical approval for this study (Protocol 733) was granted from Population Council’s Institutional Review Board in New York, with local ethical approvals from the Nigeria’s National Health Research Ethics Committee of the Federal Ministry of Health, Kano State Health Research Ethics Committee, and Ebonyi State Research Ethics Committee State Ministry of Health, in addition to Uganda’s Makerere University College of Health Sciences School of Medicine Research Ethics Committee.

## Results

### Background characteristics

Women who participated in IDIs in Nigeria and Uganda were 18 to 55 years old, were generally poor, with primary educations, or even uneducated. Most women were married; nine were separated or divorced; four never married, and one was widowed.

Fistula care seeking behavior is complex—it involves various costs, often compounded, and prolong treatment and recovery. Prevailing cost-related barrier and enabler sub-themes cluster around distinct domains (financial, income loss or opportunity cost, and transportation) (Table 2).

### Financial barriers

Generalized poverty emerged as a significant barrier to fistula repair in this study’s sample. Most women in the sample who experienced fistula are from poverty-stricken families, lack education, do not understand the necessity of regular antenatal checkups, and cannot afford antenatal and delivery care in health centers, and therefore decide upon home delivery, to seek care at health centers when complications arise, and incur higher complication-associated costs placing both themselves and their newborns at mortal risk. To meet these costs, women and their families sell property, household goods, cattle, and crops.

**Table 2:** Domains of barriers and enablers to accessing fistula care services in Nigeria and Uganda

Domain	Barrier sub-themes	Enabling sub-themes
Financial	Generalized poverty	Funding for the fistula care centers
	Cost of care at home	Pooled community funds
	Medical and non-medical out of pocket hospital costs	Vocational training or work support
	Cost for repeated medical intervention	Counseling for healing
	Unofficial fees at hospitals	
Transportation	Transportation cost	Transportation refunds—voucher
	Distance, unavailability of transportation, and prolonged travel	Family or community-facilitated transportation
	Stigmatizing behaviors in public transportation	Outreach vans/taxis/ambulances Counseled drivers
Income loss or opportunity cost	Away from small business (i.e. lost hours and productivity)	Financial support from government or family
	Away from home and children	Vocational training or work support

*“It was just the lack of money that hindered me from seeking care for eight years. We were looking for traditional treatment because of lack of money to come here…yes, no money to come here. My husband did not have any [money], and his father had none, my father had to sell some things for us to come here.”* IDI, post-repair client, Kano, Nigeria

Many families describe choosing between food and repair care, which can delay care from days to years. Persistent gender norms in Nigeria and Uganda prioritize family needs, such as food and children’s education, over women’s health (e.g. fistula care).

*“That poverty is the big thing…and lack of education…poverty is number one because it a big barrier.” IDI, woman, Masaka, Uganda*

In both countries, women are affected by the cost of care at home.

*“I have no money…I have spent all my money on soap, pampers, pieces [cloths], all this while am at home.”* IDI, client awaiting repair, Ebonyi, Nigeria

Having spent their money on self-management and intermittent visits to traditional healers, women and families lack sufficient funds for transportation and other costs for treatment at accredited facilities. One client awaiting repair in Masaka described spending up to UGX 600,000 (~US$165) to look for the “witches” who caused her fistula. Women described seeking different types of services from traditional healers, who charge varying prices.

*“Dad took me to different witches, but my husband would pay the bills. It was like that for long time until I healed…We went to several places…like Kaboyo [to] a traditional doctor and we went to Buyoga who was also a traditional healer…It was much money and the husband would pay [Interviewer: If you estimate [cost]...?] No, I can’t because the healers were different.”* IDI, client awaiting repair, Masaka, Uganda

Medical and non-medical out-of-pocket costs persist in both countries despite free repair surgery. Nigeria and Uganda mandate that hospitals bear medical and non-medical costs, namely provider remuneration (for surgery and counseling), medicines, and other ancillary expenses such as food and water. Due to funding shortages at centers and camps, clients may receive only the surgery for free but pay the non-surgical expenses—medicines, food, and water. Clients and families bear costs, regardless, of lodging, food, and drink for those accompanying them. Repair centers in Ebonyi, Nigeria, and Hoima in Uganda covered all medical and non-medical expenses, but Kano in Nigeria and Masaka in Uganda did not.

*“When I heard radio announcements and when I heard that they are going to give us transport refund and food, but they have not given us anything we have got only treatment, but for food we have catered for ourselves, and the transport has been ours.”* FGD, post-repair client, Hoima, Uganda

Women may need repeated repairs, for numerous reasons including complex or deep wound fistula that require more than one surgery. Care or counseling from providers with inadequate skills (in fistula care), at primary health care facilities or private sites ill-equipped for fistula repair often result in extra costs due to ineffective care.

*“He [provider at private clinic] did the first surgery on me free of charge, but it finally failed. He did it the second time and it failed again; it was at this time that a patient’s relation at the hospital told me about this place [fistula center].”* IDI, client awaiting repair, Ebonyi, Nigeria

Repeated surgery or medical intervention (e.g. counseling and follow up care) is sometimes necessary due to a client’s inability to maintain post-operative care. These ongoing and repeated medical interventions lead to recurring transportation and ancillary costs in both countries.

*“Maybe it was not done very perfectly, or even the mothers, the way they have been cared for, themselves. You tell a mother play sex after eight months, they just play sex because they have seen am okay and continue to leak.”* IDI, Nurse, Hoima, Uganda

Some women and families described paying an ‘unofficial’ fee, or bribe, to fistula center personnel to reduce waiting times. Providers acknowledge that corruption prevails in health facilities, especially where camps lack proper funding.

*“These women are poor and some of the facilities…they want money…The health facility doctor, to work upon you, will tell you that you give me at least UGX 500,000 and yet you do not have money—that is it, corruption among the medical workers has become rampant.”* IDI, nurse-counselor, Masaka, Uganda

### Transportation barriers

Transportation barriers can be classified as monetary, logistical (distance, mode, hours, road conditions, lack of transportation), and social (stigmatization and rude behavior experienced by women with fistula while in transit). Although fistula care is nominally free, round-trip transportation costs for at least two persons is difficult for many women, and their families, who have to borrow or sell assets to pay for transportation to fistula centers or camps.

*“What made me delay so much in the village was money. They [family members] took too long looking for it [money], and they went borrowing until they got the money. They brought me here.”* FGD, post-repair client, Hoima, Uganda

*“The little business I was doing before this sickness started, I couldn’t do it again. My husband borrowed money [for transportation], which two of us used to come to this place the first time we came; this time I came alone, but he was the one that still gave me transport fare*.” IDI, post-repair client, Ebonyi, Nigeria

Transportation costs, which vary by country, are higher in Uganda than in Nigeria. Most Ugandan women spend between 10,000 (US$3) and 90,000 (US$25) shillings for a trip to a camp, for two people (themselves and companion). In Nigeria, women from Kano spend 250 (US$0.80) to 2000 (US$6.41) Naira on transportation. Costs double or triple for women who require repeated visits, or to return to a camp or facility without routine repair services. Ugandan women and their companions travel to camps from near and far, including neighboring countries such as Rwanda and Tanzania, spending substantially on transportation.

*“For her to reach with me in Kibaale, she used 20,000 Shillings, then from Kibaale to Rakai we used UGX 6,000, from Rakai to Kyotera we used UGX 6,000 still, and from Kyotera to this place, we used 15,000 minus what we used to buy utensils…We started with UGX 40,000, and then add UGX 50,000.”* IDI, spouse, Masaka, Uganda

Distance, lack of transportation, and prolonged travel are common for clients traveling to facilities for repair. Ugandan women in remote villages walk six to 15 km or more to reach a fistula center. Women and their caregivers often use multiple modes of transportation to reach repair centers or camps due to infrequent, or no, direct transportation. Fistula clients in Nigeria are often unaware of the locations of both government hospitals and fistula repair centers. Distance and associated transportation barriers are less pronounced in Nigeria, but uneven and rough terrains are deterrents to services in both settings.

Generally, *boda* (motorcycle taxi) is the most common public transportation, in both Nigeria and Uganda, along with bus, taxi, and van, across various terrains. Some women use a family or a relative’s cycle or motorcycle, while others walk, completely or partially, to a repair hospital or camp.

*“Now where we stay in the village, we don’t have any means of transport to get us quickly to where we can access the main road where the cars are. The roads where we come from are not good, now sometimes you get a boda to take her to the main road, and yet the roads are bad, sometimes muddy with potholes, and they are serious.”* IDI, spouse, Hoima, Uganda

Women’s reliance on public transportation—especially for long distances—puts them in situations of embarrassment and discomfort about their condition and appearance, due to the foul-smelling discharge resulting from fistula. Women suffer stigmatizing and rude reactions, including refusals of service by drivers or bullying from conductors and fellow passengers. These traumatizing responses on both *boda bodas* and buses create transportation barriers to care for women living with fistula.

*“Women will fear to pass in public because people will tell her she is smelly. The boda people or taxis will refuse them…so they will go back home and sit.”* FGD, post-repair client, Kano, Nigeria

*“There is a boda guy who rode me from Kawaala to Masanafu, and I sat on his boda from church. I wet all his seat and he quarreled with my sister…never allow you sister to sit on boda again. He quarreled.”* FGD, post-repair client, Masaka, Uganda

### Opportunity cost barriers

In both countries, most women (IDI participants) who had lived with fistula were homemakers, although some maintained small family businesses or farms. After developing fistula, they describe an inability to continue these enterprises because of physical limitations, including loss of farm ownership, often a result of social separation or divorce because of their fistula condition. This loss of income represents significant opportunity costs for living with fistula. To access available free treatment, these women can face additional costs, such as employing someone else (if still working) and arranging child care, while also procuring money for the repair.

*“We are faced with serious financial challenges. The little business I was doing before this sickness started, I couldn’t do it again because this sickness made my life measurable*.” IDI, post-repair client. Ebonyi, Nigeria

### Enablers of care

Respondents emphasized the need for sustained funding of fistula centers and camps. In both countries, some centers and camps provide full financial support, with women incurring little or no out-of-pocket costs. Clients currently borrow money from extended relatives or sell household goods or property for ancillary costs such as medicine, food, water, and personal hygiene items, and there is a need for increasing the overall incomes and wealth of women who suffer from fistula, and for their families. Ugandan participants suggest that aggregated community funds could help promote access to repair care for many families. Women cannot borrow money from relatives who similarly lack financial means. Other financial enablers, such as vocational and job training for fistula clients, are discussed in the opportunity cost enablers section.

*“As we didn’t have money, only that when we heard, the announcement itself said, that those that are leaking urine and feces you should all come…and get treatment. There is free treatment and also come with the person to keep you, a caretaker, and also them their money is paid for, and also the transport that brought them will be refunded. We shall feed them and also the treatment is free. So, this forced us to come.”* IDI, family member, Hoima, Uganda

Ugandan women, as well as their family members and providers, concur that financial support for transportation, through vouchers or free ambulance services by facilities, in addition to driver-targeted counseling, for their awareness, could enable fistula clients to reach facilities more easily. All participants described their hopes for bi-directional transportation support—free transportation to and from repair facilities.

*“If we know we now have ten patients, we go and screen them. After screening them we get a passenger service van, put them in, and bring them to the center. Or we have some places where we know the buses, so we tell the patients to board those buses, and they find us on welcome [Mbarara junction] and then we pay the buses and get our patients.”* IDI, fistula surgeon, Masaka, Uganda

Familial and community support for transportation is slightly more common in Nigeria, which influences women’s decisions to seek delivery and fistula repair care at health facilities. Respondents in both countries emphasize the need to subsidize or reduce transportation fees, increase transportation frequency, and construct more vehicular roads. Nigerian and Ugandan respondents mentioned the need for building health facilities closer to their communities.

*“The center should be in rural area or close by community so that it would be easier for them to go, because to go to the town, some people are scared.”* FGD, community women, Nigeria

Income generation and social re-integration are the main priorities for women who received repair services. Participants described the importance of family financial support, as well as women’s social support groups or government—especially for divorced or abandoned women—to increase their self-sufficiency and re-payment of loans for care. Support, in the form of vocational training such as knitting, crafts, beadwork, weaving, or other applicable life skills, was advocated by all respondents. Providers suggest that counseling from community health workers along with job education would be helpful for fistula survivors’ reintegration and healing.

*“I think if they had women groups at the village levels, and these people are funded. Because, like, individually, you find some are unable to stand on their own and do anything that will benefit them, but if it is a group then they benefit.”* IDI, ANC-maternity staff, Hoima, Uganda

*“Honestly, we need assistance to help us restore our health…Because of this condition, most of us have been divorced by our husbands…Some of us don’t have fathers, just mothers, and they don’t have handwork.”* IDI, post-repair client, Kano, Nigeria

Respondents, especially women, emphasize healing and reintegration as essential components of a holistic approach that hospitals need to adopt for women’s physical, sexual, psychosocial, and economic rehabilitation after fistula repair.

## Discussion

**Fig. 1:**
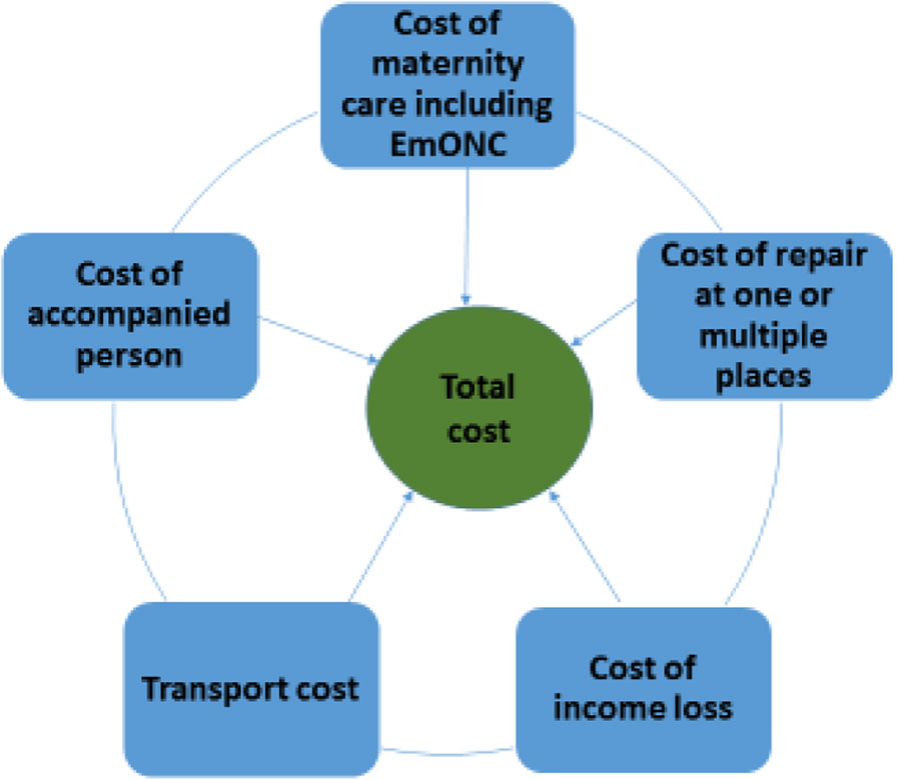
Combined and catastrophic costs of obstetric fistula care seeking

These findings demonstrate that Nigerian and Ugandan women face financial, transportation, and opportunity cost barriers to accessing obstetric fistula care (Fig. 1). The cost of obstetric fistula protracts maternity care, including emergency obstetric care, and fistula repair care costs involve expenditures at one or more hospitals, with transportation, lost income, and companion expenses. Women who suffer from multiple cost barriers often seek emergency obstetric care too late to prevent fistula [28]. In addition to managing fistula conditions at home, financially burdened and fatigued families find it extremely difficult to spend money on repair.

Our results further demonstrate that the poverty cycle exacerbates the cost burden for poor women and severely limits their abilities to seek maternal and newborn care [3, 29, 30]. The multiple personal expenses described—for ancillary care, transportation, and repeated visits—combined with opportunity costs of living with fistula burden lower socio-economic segments of society especially [22, 29]. Beyond the actual and anticipated costs delaying women’s and families’ care seeking, our data show that poverty and gender intersect and lower the priority of fistula repair, due to the fact it is generally non-fatal. Similar intersectionality is seen in Kenya, where families of lower socio-economic strata tend to maintain secrecy around a woman with fistula; studies recommend elevating women’s status as a means of bringing equity to fistula care [31, 32].

Despite similar home-based self-management conditions in Nigeria and Uganda, service costs vary, depending upon facility type (subsidized or private hospital), days at a center, nature of the fistula, and mode of transportation. In Nigeria, when women finally reach an actual free care fistula hospital, they have already spent significant amounts on transportation and prior services. Fistula clients often do not seek care because they are unaware of fistula repair center locations [22].

This study finds that fistula repair’s high financial cost affects women because of general poverty. Medical and non-medical fees, transportation costs, and occasional ‘unofficial’ fees at centers and camps generate high out-of-pocket costs for women and their families. Other conditions similarly burden the poor, as seen in a study from Ethiopia that found that not only did 27% of households experience catastrophic expenses (> 10% of household’s income) due to cardiovascular diseases, but that the poorest spent up to 34% [33]. Considering the combined costs of fistula care, which mostly begin at delivery, or right after childbirth, they are much more of a burden than other common illnesses in African countries. According to 2014 World Bank data, 72% of Nigeria’s health expenditure is out-of-pocket, while Uganda’s is 41% out-of-pocket or from private sources [34]. Ugandan women face more transportation barriers, from costs, distances, travel time, and lack of availability, than Nigerian women. Companions’ expenditures for transportation and food, frequently mentioned by Nigerian and Ugandan respondents, echo expenses described in the literature from low- and middle-income countries [5, 35]. In locations such as Nigeria and Uganda, where implementers may be unable to build new, specialized fistula centers, or administer camps in local communities, improving free transportation to accredited advanced care facilities is of great importance. In Tanzania, a program that facilitates transportation costs via mobile phone significantly increased access to fistula repair services in referral hospitals [36]. In Bangladesh, free maternity care and transportation vouchers increased women’s use of public health centers [37].

This study also examines opportunity costs, including income loss and informal loans, due to the physical condition of fistula, and proposes future programming and evaluation of women’s reintegration within community workforces—on which little global evidence exists. Our findings show that women and girls sent are away by their husbands and lose possession of assets after development of fistula [3]. Resuming societal roles as wife or mother plays important parts in women’s re-integration [38, 39]. Informal loans by relatives to women living with fistula require repayment. Nigerian and Ugandan data support the need for better post-repair life skills and job training for women’s effective re-integration to normal life [3]. The necessity of holistic care, counseling, and re-integration for the effectiveness of fistula repair programs to return women to their roles in their families and economies, through cohesive education in life skills, self-esteem, and income-generating activities, reverberates throughout the literature [3, 35].

### Limitations

Inaccuracies in self-reported information about distances to hospitals, transportation costs, and ancillary costs of care are possible limitations to the data’s quantifiability. This study was unable to obtain responses from women unable to seek repair care; additional costs and barriers experienced by these women, who are even more disadvantaged, may not be sufficiently described. The focus of the study, however, is qualitatively exploring barriers and enablers of fistula care; this approach revealed the existence of a range of cost barriers affecting care-seeking behavior for fistula repair that can be further explored. Future quantitative studies would strengthen these experientially reported figures and allow calculation of the exact financial and transportation costs women pay. This study also collected data within communities, which are often under-represented in the literature, and triangulated them with the perspectives of repair facilities and their surrounding communities, providing further credibility for our findings. Triangulation on the nature of costs and mechanisms, as well as the debilitating expenditures for women and families, suggest that the barriers described herein are indeed relevant. Another limitation of this study was a funding crisis at one hospital in each country that led to further out-of-pocket expenditures by clients.

### Implications

The study identifies relevant information for policy and programming in Nigeria and Uganda, specifically recommending that stakeholders regularly monitor and ensure sufficient funding for medicine, food, and ancillary costs at hospitals and camps offering fistula repair. It further suggests that community funds, wherever possible, should be aggregated to enable women to borrow and repay loans to access repair care. Given the low relative burden fistula compared to other health conditions, national financial risk-pooling in accordance with notions of universal health coverage are worth considering in both Nigeria and Uganda [36]. Transportation support through cash, refunds, or vouchers, to both women and their companions, can enable access by poor and hard-to-reach clients to the limited times and places for fistula care. Similar to risk-pooling for direct services, incorporating transportation costs into universal health coverage schemes suggest a comprehensive approach for conditions like fistula. Community awareness of fistula repair centers and available services must be improved by strengthening local information-sharing mechanisms. Finally, opportunity costs suffered by women who have had fistula must be reduced by increasing vocational training opportunities for post-repair clients’ workforce re-entry.

## Conclusion

The total cost of fistula repair often reveals an inability by impoverished women to prioritize spending on treatment over other household expenditures. Although fistula is not immediately life-threatening, it destroys women’s social, economic, and family lives. The stress, depression, stigma, and vulnerability of living with fistula each demand women’s early treatment, counseling, and re-integration to society. It is critical to support women’s workforce re-entry and mitigation of income loss due to fistula, with the broader economic development of these women’s families and communities. We recommend innovative approaches for financing transportation, improving available information on repair centers and rehabilitation, and addressing the multi-dimensional cost barriers described in this study.
